# Did Premodern Wars Impact Sex Ratios at Birth? The Case of 19th-Century Basque Country

**DOI:** 10.1007/s12110-025-09496-8

**Published:** 2025-07-09

**Authors:** Francisco J. Marco-Gracia, Francisco J. Beltrán Tapia, Grażyna Liczbińska

**Affiliations:** 1 Department of Applied Economics and Instituto Agroalimentario de Aragón (IA2), Universidad de Zaragoza, Zaragoza, Spain; 2 Norwegian University of Science and Technology, Trondheim, Norway; 3 Center for Economic Policy Research (CEPR), London, UK; 4 Institute of Human Biology and Evolution, Faculty of Biology, Adam Mickiewicz University, Poznań, Poland

**Keywords:** Sex ratios at birth, Maternal stress, War, Parish registers, Basque country, 19th century

## Abstract

This study examines the impact of pre-moder wars on sex ratios at birth. Specifically, it investigates whether wars involving premodern weaponry and more limited destruction increased stress for pregnant women such that the resulting hormonal changes in their bodies may have led to a decrease in the ratio of live-born boys to girls. To address this question, we analyzed all baptismal records from the Basque Country during the 19th century, encompassing nearly 1.2 million individuals. Our findings confirm that the First Carlist War (1833–1840) exercised the most significant demographic impact on the region. However, all wars resulted in variations in demographic behavior, reducing in sex ratios at birth by around 1%. This effect is even more pronounced when focusing on the main conflict zones, where sieges and major battles took place, leading to an impact of 3.2%. The effect was not only immediately visible in sex ratios at birth but became particularly evident during the first three months after the events. Furthermore, prolonged battles were found to have the most substantial influence, with an average effect during the three months after the battle of almost 7% on sex ratios at birth. In conclusion, our study demonstrates that premodern wars reduced sex ratios at birth.

**Supplementary Information:**

The online version contains supplementary material available at 10.1007/s12110-025-09496-8.

## Introduction

The effects of human conflicts on human nature have been a subject of extensive debate for decades (Fromm, 1992; Grossman, 2018). One approach to understanding this relationship is through its impact on birth rates. The proportion of boys to girls at birth, commonly referred to as the sex ratio at birth (SRB), serves as a critical demographic indicator, reflecting both population dynamics and potential fertility patterns (Kemkes, 2006, p. 806). In natural environments where access to nutrition is adequate and stress levels are not excessively high, approximately 105–107 boys are born for every 100 girls (Cavalli-Sforza & Bodmer, 1971; Orzack et al., 2015). This surplus of male births is a well-documented phenomenon, functioning as a biological strategy to offset higher male mortality rates during infancy and early childhood (Byrne & Warburton, 1987; Hassold et al., 1983; Marco-Gracia & Beltrán Tapia, 2021).

Adverse maternal conditions, however, influence the sex ratio at birth by increasing intrauterine and birth mortality, which would especially affect males fetuses (James, 2006; James and Grech 2017; Long et al. 2021; Morse and Luke 2021). A particular strand of the literature stresses how adverse cirumstances (temperature, chemical pollutants, or even conflicts) reduce the SRB because male fetuses are more vulnerable to miscarriages (Bruckner and Catalano 2018). Alternatively, Trivers and (Willard 1973) argue that during times of prosperity, natural selection favours mothers who give birth to male foetuses because they are not under severe stress. In times of stress, by contrast, natural selection mechanisms decrease male births by reducing the number of conceptions and increasing the proportion of spontaneous abortions of male foetuses, ultimately leading to a lower sex ratio at birth. Existing literature indicates that wars negatively affect the SRB, typically resulting in a decline in the proportion of male births. For example, the brief 10-day war in Slovenia during the Yugoslav Wars demonstrated that acute psychological stress led to a measurable decrease in SRB six to nine months later (Zorn et al., 2002). Similar reductions in SRB have been observed during World War II in Austria, Belgium, Denmark, England and Wales, France, Germany, the Netherlands, and the United States (Bethmann & Kvasnicka, 2012).

However, the relationship between warfare and SRB is not universally consistent. For instance, De Jastrzębski (1919) observed an increase in SRB during World War I in Ireland, Australia, New Zealand, and Finland (see James, 2009). Similarly, Bethmann & Kvasnicka (2012) documented a rise in SRB in Germany during World War II. In Poznań, Poland, a comparison between pre-WWII and WWII periods revealed higher SRB values during the war (109 boys per 100 girls) than in the prewar era (97 boys per 100 girls; Liczbińska & Kralik, 2020, 2022). According to Liczbińska and Marco-Gracia (2024), shorter gestational age (GA) may have played a crucial role in the higher number of live-born boys compared to girls during WWII, since pregnancies tended to end pre-term during the war. As delivery approaches in the later stages of pregnancy, various factors can affect survival, leading to a decrease in the male-to-female sex ratio (Orzack et al., 2015). Increases in SRB during and after both World Wars were also noted in Austria, Belgium, Bulgaria, England, France, Germany, Hungary, Italy, Romania, and South Africa (Bernstein, 1958; Bethmann & Kvasnicka, 2012; Russell, 1936). Between 1939 and 1945, the SRB rose in the USA, Canada, and England (Myers, 1949). Bromen and Jockel (1997) further confirmed an increase in SRB in Germany during the Second World War, including in both East and West Germany. In contrast, no significant changes in SRB were reported during the Spanish Civil War and its aftermath (1936–1941; Grafelman & Hoekstra, 2000). In Tajikistan, after the dismantling of the Soviet Union and the subsequent civil war, the sex ratio at birth increased significantly from 104.6 before the war to 106.9 during the war (Hofmann et al., 2010). Similarly, in the civil war following the dismantling of Yugoslavia in 1991–1995, the sex ratio increased in Bosnia-Herzegovina from 106.6 in the pre-war period to 109.6 and then declined to 106.2 in the post-war period (Polasek, 2006).

The variability in findings underscores the lack of a definitive answer regarding the impact of wars on SRB. Furthermore, much of the existing literature focuses on 20th-century conflicts characterized by the use of modern weaponry with immense destructive capabilities. As James (2009) emphasizes, there remains limited understanding of the effects of premodern wars on SRB, despite the ubiquity of conflict throughout human history. Historical examples provide mixed evidence: Dusing (1884), cited by Lawence (1941), reported an increase in SRB in Sweden during the Russo-Swedish War (1789–1790). While Kemkes (2006) observed a decline during the French Revolutionary Wars (1792–1797), Nichols (1906) found no significant changes in the same period. Similarly, studies of the Franco-Prussian War (1870–1871) found no notable alterations in SRB (Nichols, 1906; Gini, 1908; Nixon, 1916; Savorgnan, 1921). This raises the question of whether historical wars lacked the destructive intensity required to affect human biology significantly.

To explore the relationship between warfare and SRB, it is crucial to examine the underlying mechanisms linked to maternal well-being. A decrease in SRB (an increase in the proportion of female births) is often associated with environmental stressors that trigger physiological responses, including hormone secretion, which can impact fetal development and increase the likelihood of spontaneous miscarriage (Byrne & Warburton, 1987; Hobel et al., 1999). Stress has been shown to reduce sperm quality and motility, further contributing to declines in male births (Fukuda et al., 1996; Zorn et al., 1999). Male fetuses, being more vulnerable to prenatal stress, are miscarried more frequently, leading to a higher proportion of female newborns (Torche & Kleinhaus, 2012). Other stress-inducing events, such as natural disasters (Fukuda et al., 1998), floods (Lyster, 1974), and economic crises (Catalano, 2003), have similarly been linked to reductions in SRB. Wars introduce a myriad of psychological stressors, including the threat of death, loss of family and property, chronic malnutrition, exposure to infectious diseases, and deteriorating socioeconomic conditions (Liczbińska et al., 2019; Fihel, 2020).

This study aims to investigate whether 19th-century wars generated sufficient stress to influence SRB in the Basque Country, a Spanish region that was a central theater of civil conflict throughout the 19th century, particularly during the First Carlist War. The availability of comprehensive parish records for the entire region (including around 1.2 million baptisms), combined with detailed accounts of historical conflicts, offers a unique opportunity to use this region as a natural historical experiment. By employing descriptive statistics and regression models, this study seeks to uncover the impact of these conflicts on human biology. The availability of individual-level microdata enables controlling for critical factors such as birthplace and maternal circumstances, ensuring a nuanced analysis of this complex phenomenon.

## Area, Data and Methods

### Area

**Fig. 1:**
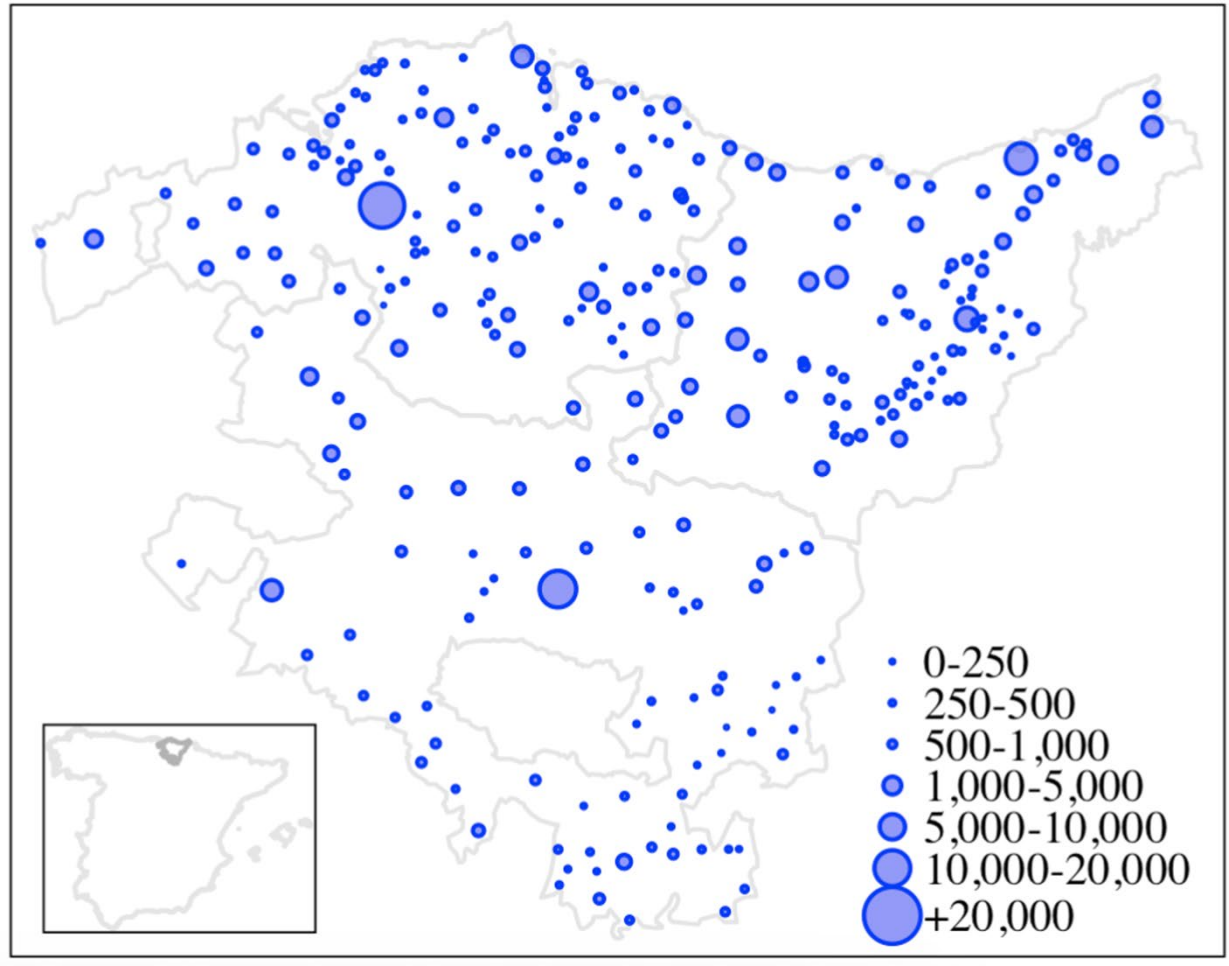
Location of the Basque Country and local population, 1860. Source: Own rendering

The Basque Country, situated in northern Spain along the western Pyrenees and the Bay of Biscay (see Fig. 1), comprises three provinces: Álava, Gipuzkoa, and Bizkaia.^1^ At the start of the 19th century, the Basque Country’s economy was primarily agrarian, with agriculture, fishing, and artisanal crafts forming the backbone of economic activity. The social structure was deeply rooted in traditional practices and governed by the *fueros*—a set of regional privileges that granted the Basque provinces partial autonomy, including exemptions from certain taxes and military service (Kurlanski, 2000). By the mid-19th century, the region began to undergo significant industrialization. This transformation was fueled by an abundance of natural resources, particularly iron, as well as the development of infrastructure, such as ports and railways, coupled with foreign investment and technological progress. These changes gave rise to a prominent entrepreneurial class, including influential families like the *Ybarras* and *Chávarris*, who played a central role in establishing industries in sectors such as shipbuilding, steel production, and finance. This industrial boom turned the Basque Country into an economic leader in Spain, contributing substantially to the nation’s industrial output by the end of the century (Larrinaga, 2018). The rapid growth of industry also spurred urbanization, particularly in cities like Bilbao and San Sebastián, which saw dramatic population increases.

Throughout the 19th century, the Basque Country’s population grew significantly, reflecting broader demographic trends in Spain. At the beginning of the century, the region was home to roughly 600,000 people, predominantly concentrated in Gipuzkoa and Bizkaia, with Álava having a smaller share. By the mid-century, the population had reached approximately 800,000, driven by improved agricultural practices, urban expansion, and migration from rural areas (see Fig. 1). Bizkaia, in particular, experienced remarkable population growth due to its industrialization, with Bilbao emerging as a key urban center. By the late 19th century, the population had exceeded 1.3 million, with Bizkaia remaining the most densely populated province, followed by Gipuzkoa and Álava. Additionally, emigration, particularly to the Americas, and economic transformations linked to the Industrial Revolution also shaped demographic patterns.

However, the century was not without turmoil. The Basque Country endured significant disruptions during the Napoleonic/Peninsular War (1808–1814) and the Carlist Wars (1833–1839, 1872–1876), which caused numerous deaths (an estimated 40,000 to 80,000 deaths occurred in the Basque Country during the Carlist Wars) and inflicted widespread damage on infrastructure, trade, and the regional economy (see Sect. 3 for more details). The abolition of the *fueros* after the Carlist Wars further weakened the traditional political and economic autonomy of the Basque provinces. This loss of autonomy was a key factor in the emergence of Basque nationalism in the latter part of the century (Clemente, 1982).

### Data

The data for this research were extracted from baptism, marriage, and death records from all Basque parishes. Initially, all available records were centralized in the Diocesan Archives of each of the three provinces. In the early 21st century (concluding in 2003), the regional government of the Basque Country digitized all available records dating from the 15th century (the earliest being from the parish of Antzuola, Gipuzkoa, in 1498) up to the year 1900. These digitized records have been made publicly accessible online for genealogical research^2^ and are also accessible to researchers through the region’s general civil archives. The data encompass 100% of the localities, although occasional issues with preservation or incomplete records may arise. For this study, we have exclusively utilized records from the 19th century, specifically between 1800 and 1899. Table 1 presents the number of births, baptisms, and deaths for each province, which we have accessed and analyzed at an individual level.^3^ The sex of individuals was determined based on their given baptismal name.^4^

**Table 1:** Number of observations by type and province, Basque Country, 19th century

Province (Capital)	Baptisms	Marriages	Deaths
Araba (Vitoria)	342,351	79,552	257,530
Bizkaia (Bilbao)	593,697	150,155	392,983
Gipuzkoa (San Sebastian)	488,423	117,290	323,470

Source: Complete parish registers of the Basque Country

Baptismal/birth^5^ records usually include the name of the child, date of birth and baptism, names and surnames of the parents and grandparents and their places of origin (often with their occupations), and the names of godparents, reflecting familial and social networks. Marriage records document the names, ages, marital status, and residences of the bride and groom, along with the names of their parents. Death records commonly report the deceased’s name, age, occupation, marital status, cause of death, and place of burial, occasionally including information on surviving family members.

In Table 2, we present the population size of the Basque provinces, the region as a whole, and its main cities between 1787 and 1900. As can be seen, the region’s population more than doubled in just over 50 years. This growth reflects the process of industrialization experienced throughout the century. To estimate the population for the intervening years, we used interpolation methods.

**Table 2:** Population of the Basque Country: provinces, main towns, and the region as a whole (1787–1900)

Province/Town	1787/9	1842	1857	1860	1877	1887	1897	1900
Araba	60.000	70.134	96.398	97.934	93.538	92.915	94.622	96.385
Bizkaia	100.000	112.814	156.493	162.547	167.207	181.845	191.822	195.850
Gipuzkoa	120.000	98.697	160.579	168.705	189.954	235.659	290.222	311.361
**BASQUE COUNTRY**	280.000	281.645	413.470	429.186	450.699	510.419	576.666	603.596
Bilbao	11.000	10.234	17.923	17.969	32.734	50.772	74.093	83.306
San Sebastian	8.000	10.036	15.911	14.111	21.355	29.047	35.975	37.812
Vitoria	7.500	9.553	18.710	18.728	25.039	27.660	30.514	30.701

Note: For the year 1787/89, the figures are approximate estimates based on the Floridablanca Census, which does not include data on children under the age of seven

Source: *Instituto Nacional de Estadística* (INE)

### Methods

We conducted regression analyses to examine how sex ratios at birth behaved before, during and after wartime in the Basque Country during the period of study. Logistic regression models were employed to analyze the relationship between the likelihood of a newborn being male and various explanatory variables, including period of birth (that is, whether the individual was born during a conflict or peace), season of birth (we distinguish by season of birth, as in a pre-industrial world—subject to food shortages and epidemiological crises—seasonality may help explain certain fluctuations in sex ratios at birth.), locality (we know the exact birthplace of each individual, allowing us to control for geographic, climatic, and/or cultural factors associated with each location), year and year squared (due to the potential effect of the passage of time on the observed trends.)^6^ The dependent variable was categorical, coded as 0 for females and 1 for males. Four sets of logistic regression models were developed (Tables 2, 3, 4 and 5), which can be represented as follows:

$$\:{P}_{i}\:\left(boy=1\right)=\frac{1}{1+{e}^{-(\alpha\:+{\beta\:}_{1}*{Period}_{1i}+{\beta\:}_{2}*{Season}_{2i}+{\beta\:}_{3}*{Locality}_{3i}+{\upepsilon\:})}}$$

In the regression equation, the dependent variable P (boy = 1) represents the outcome for an individual i, where β_n_ is the parameter, X_n_ (Period/Season/Locality) and ε represents the error term. Additionally, to evaluate the combined significance of the independent variables, we performed a likelihood ratio test, confirming that the test statistics follow a chi-square distribution. This result supports the explanatory power of the model.

## Historical Context: The Napoleonic and Carlist Wars

Throughout the 19th century, the Basque Country experienced four major conflicts, significantly shaping its socio-political and economic landscape (Juaristi, 2013). The first of these was the Peninsular/Napoleonic War (1808–1814), fought between Napoleonic France and the allied forces of Spain, Britain, and Portugal. Due to its strategic location along the border with France, the Basque Country became a focal point for both occupying forces and local resistance. The war severely disrupted trade and agriculture while destroying critical infrastructure. Basque guerrilla fighters, leveraging the region’s rugged terrain, played a pivotal role in resisting French occupation. This period also heightened tensions within Basque society, as liberal policies introduced by Napoleon—and later upheld by the Spanish monarchy—challenged the traditional regional privileges, or *fueros*, that had long defined Basque autonomy (Montero, 1995). This period also heightened tensions within Basque society, as new policies introduced by Napoleon—and later upheld by the Spanish monarchy—challenged the traditional regional privileges, or *fueros*, that had long defined Basque autonomy (Montero, 1995).

Key events of the Napoleonic War in the Basque Country included the Siege of San Sebastián (July-August 1813), where allied forces bombarded and captured the city after fierce fighting. The siege resulted in extensive destruction, including a devastating fire that consumed much of San Sebastián and caused approximately 1,000 civilian casualties. Another major event was the Battle of Vitoria (June 21, 1813), where British, Spanish, and Portuguese forces under the Duke of Wellington decisively defeated French troops led by King Joseph Bonaparte. Finally, it is important to highlight the decisive Battle of the Bidasoa, fought on the Spanish-French border on October 7, 1813. This victory ended the French occupation in northern Spain.

In the aftermath of the Peninsular War, ideological divisions in the region led to the outbreak of the Carlist Wars (1833–1876). These conflicts arose from disputes over the Spanish throne and were characterized by resistance to centralizing policies and the defense of regional autonomy and traditional values. The First Carlist War (1833–1839), sparked by the succession of Queen Isabel II over her uncle Don Carlos, saw fierce fighting in the Basque Country, Navarre, and Catalonia. The conflict resulted in widespread human loss, with an estimated 100,000 fatalities, including civilians, due to combat and guerrilla warfare. Notable events in the Basque Country included the Battle of Oriamendi (1837), where Carlist forces successfully repelled a pro-Queen Elizabeth II (central government) offensive near San Sebastián, bolstering morale and emphasizing their commitment to preserving Basque privileges. The Siege of Bilbao (1835, 1836–1837) also underscored the Carlist effort, though their repeated attempts to capture the city ultimately failed. Notable events of the conflict also include the siege of the city of Vitoria on March 16, 1834; the engagement at Chinchetru on October 27, 1834; and the skirmish that occurred a day later, on the eastern outskirts of Vitoria. Other significant moments include the Battle of Ormáiztegui on January 3, 1835; the siege of the small town of Ordizia from May 25 to June 3, 1835; the Battle of Mount Arlaban (January 16–18, 1836); the siege of San Sebastián, the second-largest city in the Basque Country, on May 5, 1836; the battle following the siege of Bilbao along both sides of the estuary connecting the city to the sea throughout December 1836; the Battle of Andoain on September 14, 1837; the Battle of Irún near the end of the war on May 17, 1838; and, finally, the noteworthy Battle of Peñacerrada from June 20 to 22, 1838.

The Second Carlist War (1846–1849), though shorter and less intense, was driven by the failed marriage negotiations between Isabel II and the Carlist pretender Carlos VI. Centered mainly in Catalonia, this conflict caused regional disruptions and an estimated 20,000 deaths but had a limited impact on the Basque Country (no noteworthy battles to report).

The Third Carlist War (1872–1876) emerged during Spain’s political instability following the Glorious Revolution (1868) and the brief reign of King Amadeo I. Carlist forces, led by Carlos VII, once again found strong support in the Basque Country, Navarre, and parts of Catalonia. The prolonged conflict caused approximately 50,000 deaths and culminated in a decisive Carlist defeat. This loss marked the end of the Basque provinces’ *fueros* and regional autonomy, which further fueled the emergence of Basque nationalism in the late 19th century. During the Third Carlist War, in the Basque Country, the most significant battles took place in Bilbao and its surroundings. The conflict began with a Carlist siege aimed at capturing Bilbao, the major regional capital, which lasted from February 21 to May 2, 1874. Following their failure to seize the city and subsequent defeat, the fighting shifted to the outskirts of Bilbao, near the coast. Here, central-government forces sought to reclaim Carlist-held territories in a protracted struggle, including the actions at Abanto and Ciérvana from May 25 to 28, 1874, as well as the naval engagement and bombardment of Ondarroa on June 26 and 27, 1874. Later in the war, on February 5, 1876, the Battle of Abadiano also took place within the province of Biscay.

**Fig. 2:**
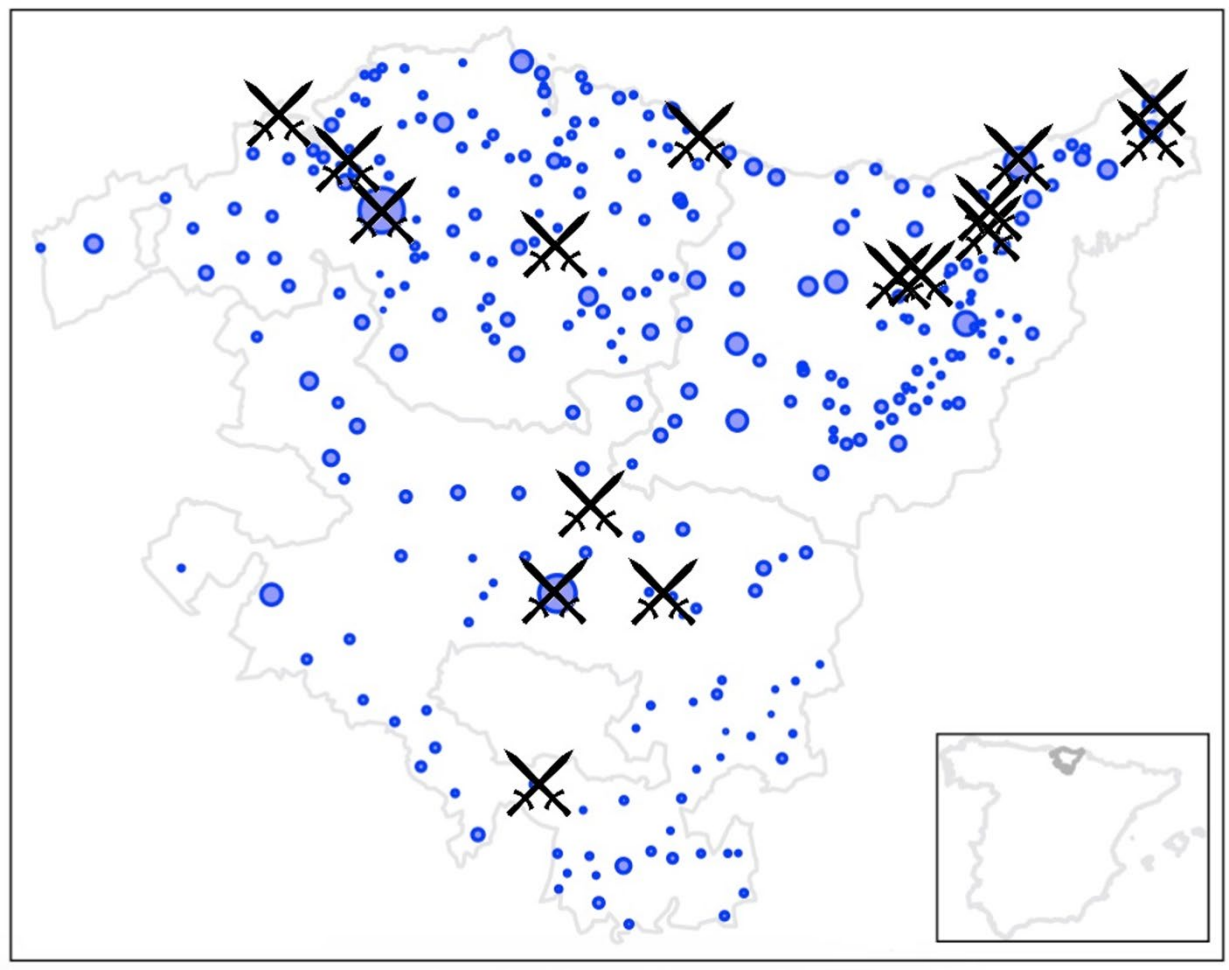
Location of the main battles of the Napoleonic War and Carlist Wars. Note: As discussed in the text, the major battles correspond to the First Carlist War. The principal engagements of the Napoleonic Wars were concentrated in Vitoria (to the south) and, more prominently, near the French border (to the north). In contrast, there were no significant battles during the Second Carlist War, while all the notable battles of the Third Carlist War were concentrated in Bilbao and its estuary (in the northwest). Source: Own rendering

A detailed map of these major 19th-century battles and events in the Basque Country, including their dates, is provided in Fig. 2. This visual reference complements the analysis by highlighting the key military episodes that shaped the region during this tumultuous century (Clemente, 1982, 2011; Oury, 1995).

## Evolution of Demographic Behavior

**Fig. 3:**
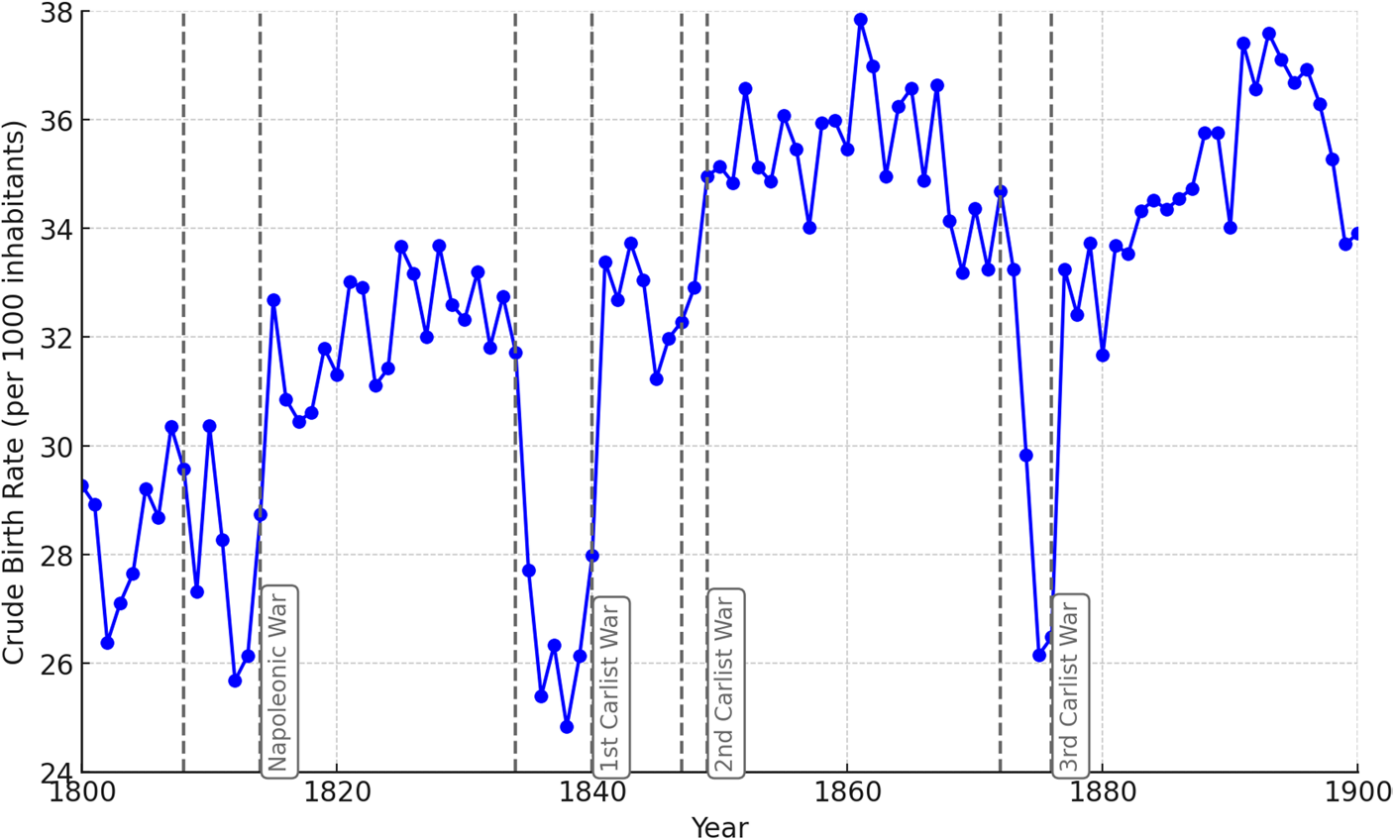
Crude Birth Rate (CBR), Basque Country, 19th century. Source: Complete parish registers of the Basque Country

Before analyzing the sex ratios at birth, the primary variable of this study, we first examine the behavior of key demographic variables during the war periods to understand the impact of each conflict and the patterns that emerge. Figure 3 illustrates the trends in the Crude Birth (Baptism) Rate throughout the 19th century. A clear decline in fertility is observed during the Napoleonic War, the First Carlist War (which saw the most significant drop in fertility), and the Third Carlist War, while the Second Carlist War appears to have had no notable impact.

The decline in fertility can be explained by several factors: the biological and economic stress associated with wars and famines, which increases the likelihood of spontaneous abortions (Catalán and Lanza, 2015), miscarriages or stillbirths; the mobilization of young men for conflict, leading them to leave their homes; the lengthy separation of many spouses and a decline in the number of newlyweds; and/or a conscious decision to limit fertility due to uncertainty about the future. In any case, Fig. 3 provides clear evidence of the impact of different conflicts and highlights the periods during which the most significant demographic changes—and potentially shifts in sex ratios—can be expected (Catalán, 2022, 2023).

An alternative explanation for the decline in fertility could be related to disruptions in the registration of baptisms during the conflict, i.e., loosening of organizational efficiency and obstacles due to wars and other disasters and/or negligence at the time. However, we have no evidence to suggest that this was a decisive factor. On the contrary, based on the combined records of births, marriages, and especially deaths, we can confirm that most parish priests remained in their local communities and continued to administer the sacraments. Baptism was essential for attaining salvation—particularly in a conservative society like Spain’s, and even more so in the Basque Country.

There also does not appear to be a widespread disappearance of baptismal records during the wars. This may have been a somewhat more relevant issue during the Napoleonic Wars, when soldiers occasionally destroyed parish records in isolated cases. Nevertheless, none of this seems to be a major factor in explaining the observed changes in fertility, and even less so in the shifts in sex ratios.

One factor more plausibly linked to the decline in fertility in the months following the outbreak of war is the conscription of young and adult men, especially given the proximity of the conflict. This may have had reduced the number of men in the marriage market and on the number of future fathers.

**Fig. 4:**
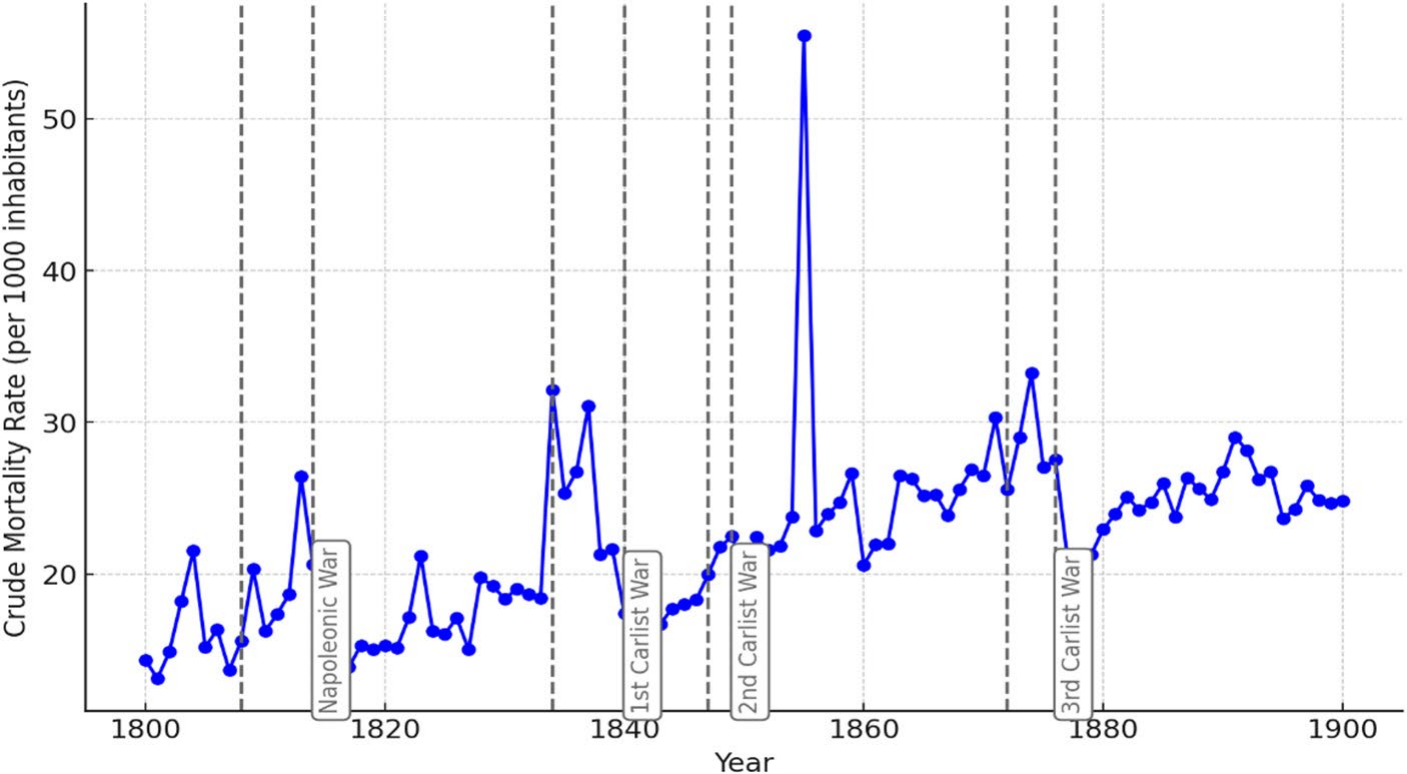
Crude Mortality Rate (CMR), Basque Country, 19th century. Source: Complete parish registers of the Basque Country

Figure 4 illustrates the response of another biologically influenced variable to 19th-century conflicts: the evolution of mortality. The figure clearly shows that the most significant increases in mortality compared to preceding periods occurred during the Napoleonic War and, most notably, during the First Carlist War. During this latter conflict, the Crude Mortality Rate increased by 50% for most of the period. In contrast, no impact is observed during the Second Carlist War, and during the Third Carlist War, the increase compared to the previous five years was modest, at just 10%. The prominent spike in the year 1855 corresponds to a cholera epidemic (Echevarría-Abascal, 1994).

The combined interpretation of Figs. 3 and 4 indicates that the First Carlist War had the most severe demographic and biological consequences in the region. The Third Carlist War and the Napoleonic War had a significant but somewhat lesser impact, while the Second Carlist War showed no measurable effects.

**Fig. 5:**
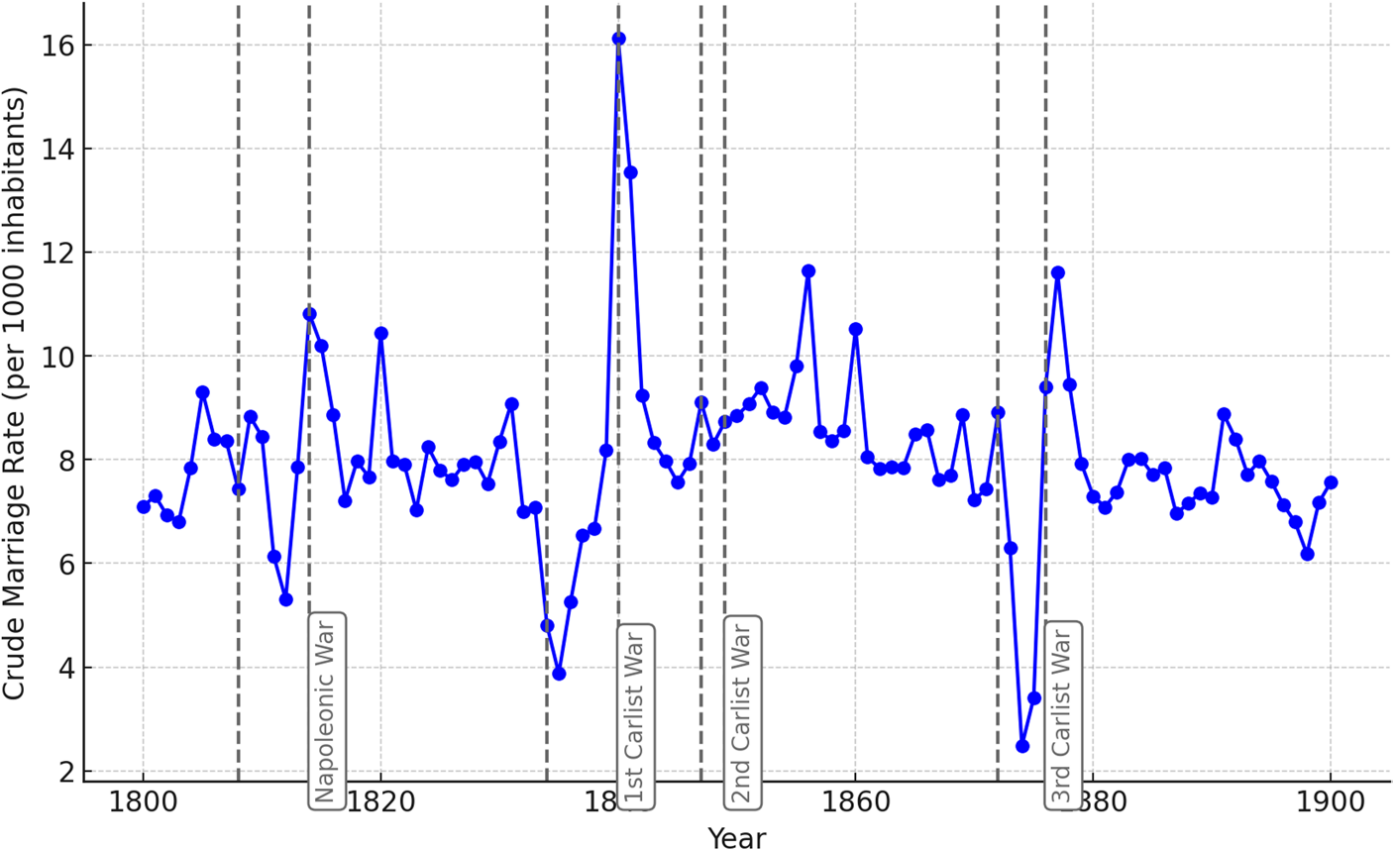
Crude Nuptiality Rate (CNR), Basque Country, 19th century. Source: Complete parish registers of the Basque Country

Figure 5 examines the trends in nuptiality within the study area using the Crude Nuptiality Rate (CNR). Unlike the previously analyzed variables, marriage rates reflect human agency, as they usually represent deliberate decisions made during periods of stress and conflict.

Figure 5 shows that nuptiality declined by approximately 25% during the Napoleonic War compared to prior years, by 50% during the First Carlist War, and nearly 75% during the Third Carlist War, with virtually no effect observed during the Second Carlist War. This might suggest that the Third Carlist War had the greatest intensity. However, when considering Figs. 3 and 4 alongside the existing literature on the impact of 19th-century wars, it becomes evident that the pronounced effect of the Third Carlist War is likely tied to human agency. Couples likely had a greater capacity to postpone marriages during this period, amplifying the observed decline in nuptiality. The high number of casualties, particularly among young men, may have resulted in a gender imbalance, leaving fewer men available for marriage and consequently reducing the overall demand for marriage (Battistin et al., 2021). Thus, the increase in marriages after wars may have been due to increased competition among women for the remaining men. The high levels of uncertainty and risk associated with war may have made people more reluctant to commit to long-term relationships, such as marriage. The fear of losing a partner who was a breadwinner, supporter and source of security may also have discouraged marriage. Additionally, individualism and secularization likely increased throughout the 19th century (Lee, 2017; Vilches, 2023), suggesting a possible growing role of human agency over time and, especially, during the Third Carlist War.

This postponement of marriages could also influence fertility trends shown in Fig. 3, exaggerating the direct impact of the conflict. The reduction in fertility was partially mediated by the decline in new marriages rather than being solely attributable to the direct stress of the war.

Figures 3 and 4, and 5 together offer an overview of the evolution of demographic behavior in the Basque Country. Figure 3 shows a steady increase in the crude birth rate throughout the 19th century. In a society undergoing advanced industrialization—well above the Spanish average—the value placed on children grew over the course of the century. This likely contributed to rising fertility levels, along with a decline in the average age at marriage (García-Sanz, 1988). Similarly, as shown in Fig. 4, the crude mortality rate also increased over the century. This trend was largely due to persistently high infant and child mortality, which only began to decline in the final third of the century—and even then, with difficulty, particularly in overcrowded urban environments brought about by industrialization. By contrast, Fig. 5 reveals a very slight, almost imperceptible decline in the crude nuptiality rate. This may be related to the influx of male laborers employed in industry, many of whom eventually returned to their places of origin to form families. As a result, nuptiality rates were not necessarily boosted by industrial growth.

## The Impact of the War on Sex Ratios at Birth

**Fig. 6:**
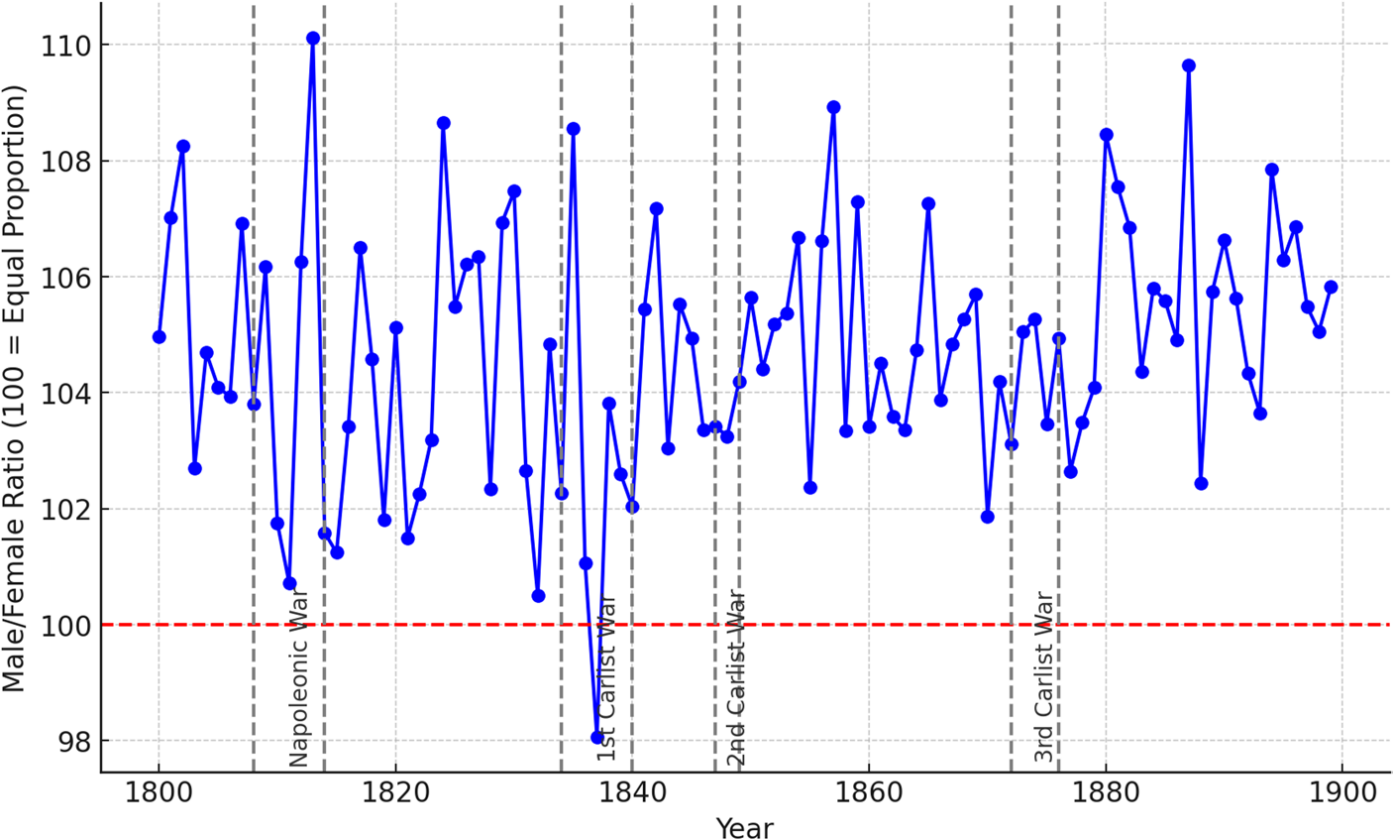
Evolution of sex ratios at birth in Basque Country, 19th century. Source: Complete parish registers of the Basque Country

Figure 6 illustrates the annual evolution of sex ratios in the study area, with the years of war highlighted, as in previous cases. During the Napoleonic Wars, a significant decline in sex ratios at birth was observed, reaching notably low levels with an almost equal number of boys and girls born during the early years of the conflict. However, this trend reversed towards the end of the war, culminating in the highest sex ratios at birth recorded in the nineteenth century. In any case, it is evident that the conflict with the most significant impact on sex ratios at birth in the Basque Country was the First Carlist War. This is the only period during which sex ratios fell below 100 (indicating more girls than boys were born). We can suppose that the reason might have been the early intrauterine death caused by psychological stress or starvation induced by the war, to which male embryos seem to be more susceptible compared to female embryos (Vatten & Skjaerven, 2004).

In contrast, the Second and Third Carlist Wars did not produce significant changes in sex ratios at birth. In order to have a better understanding of the effect of war on SRB, the analyses below link the temporal and spatial information on these conflicts with the individual-level records extracted from parish registers.

During the Napoleonic Wars, a significant decline in birth rates was observed, reaching notably low levels with an almost equal number of boys and girls born during the early years of the conflict. However, this trend reversed towards the end of the war, culminating in the highest sex ratios at birth recorded in the nineteenth century. In contrast, the Second and Third Carlist Wars did not produce significant changes in sex ratios at birth. In order to have a better understanding of the effect of war on SRB, the analyses below link the temporal and spatial information on these conflicts with the individual-level records extracted from parish registers.

### War Years

Firstly, we will examine whether the years during which the various wars of the 19th century occurred in the Basque Country significantly altered sex ratios at birth.^7^ To this end, Table 3 presents an analysis of the probability of being born male using three logistic regression models. The dependent variable takes the value 1 if the individual is baptized as male and 0 if baptized as female. In addition to the basic relationship between sex and period (Model 1),^8^ Model 2 incorporates birth seasonality and Model 3 accounts for fixed effects associated with geographic factors.^9^

The results align closely with the discussions presented in previous sections. During all wars, the probability of female births slightly increased compared to the reference period. However, only the First and Third Carlist Wars show statistically significant results, with coefficients around or slightly exceeding 0.020. Notably, in Model 3, where geographic context is also controlled for, the significance of the Third Carlist War disappears. This suggests, as observed in Fig. 6 regarding the evolution of sex ratios in the Basque Country, that the conflict with the greatest impact on the region was clearly the First Carlist War. The variables year and year squared do not yield significant results, as anticipated from the figure illustrating the evolution of sex ratios at birth.

Similar patterns emerge when using alternative peace periods as reference points, though with varying coefficients. Generally, years of conflict were associated with lower-than-usual sex ratios, likely as a result of maternal stress—a hypothesis we will explore further in the next section. Among these wartime periods, the destructive impact of the First Carlist War stands out most prominently.

**Table 3:** Probability of being registered at birth as a boy in Basque County, 19th c

Dependent variable: Sex (male = 1)		(1)	(2)	(3)
War period	Before Napoleonic War	(ref.)		
	Napoleonic War	-0.005 (0.01)	-0.004 (0.01)	-0.012 (0.01)
	First Carlist War	-0.026** (0.01)	-0.025** (0.01)	-0.041** (0.01)
	Second Carlist War	-0.015 (0.02)	-0.015 (0.02)	-0.026 (0.02)
	Third Carlist War	-0.030** (0.02)	-0.030** (0.02)	-0.030 (0.02)
	Interwar periods	-0.014 (0.01)	-0.013 (0.01)	-0.019 (0.01)
	After Third Carlist War	-0.015 (0.02)	-0.016 (0.02)	-0.025 (0.02)
Season of birth	Winter	(ref.)		
	Spring		-0.021*** (0.01)	-0.016** (0.01)
	Summer		0.005 (0.01)	0.007 (0.01)
	Fall		0.002 (0.01)	0.003 (0.01)
Control Year		Yes	Yes	Yes
Control Year squared		Yes	Yes	Yes
Locality Fixed Effects		No	No	Yes
	Sample size	1,209,058	1,204,861	1,204,860

Source: Complete parish registers of the Basque Country

Notes: *se* denotes robust standard error. * Statistical significance at 10% level, ** at 5% level. *** at 1% level. The same analyses, excluding the variables *year* and *year squared*, can be found in Table A.1 of the Appendix. For simplicity, the intercept is not reported

### The Place of Conflict

**Fig. 7:**
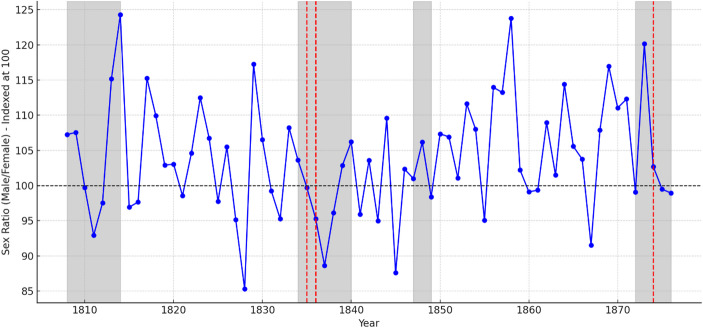
Evolution of sex ratios in the city of Bilbao: the map highlights the wartime periods (in gray) and the three sieges endured by the city (in red). Source: Complete parish registers of Bilbao

The next question we address is whether the impact of war on sex ratios at birth was widespread across the entire Basque Country or primarily concentrated in areas where the main battles and sieges took place.^10^ Fig. 7 illustrates the evolution of sex ratios in Bilbao, the most populous city in the region (17,969 inhabitants in 1860 and 83,306 in 1900). Bilbao also endured the most severe sieges during the conflicts, specifically from June to July 1835, October to December 1836, and again from February to May 1874. While the patterns of sex ratios in the figure are not entirely straightforward, it is evident that sex ratios consistently declined in the year following each siege experienced by the city.

Based on these observations, we decided to investigate the impact of battles and sieges on the broader set of localities that endured the most intense wartime actions, focusing on both the year in which the events occurred and the year that followed. To explore this issue systematically, we classify our locations into three groups: localities that experienced the most significant conflicts of the 19th-century wars in the Basque Country (as listed in Sect. 3 of the *Historical Background*), the rest of the region during wartime (including these same localities when battles were not occurring within their territory), and the region as a whole during peacetime. Furthermore, we consider whether the effect was immediate or whether it requires accounting for a lag period. Additionally, we analyzed the effects on the specific day of the events and in the subsequent three months, six months, and nine months (corresponding to the duration of a full pregnancy). Table 4 replicates the full model from Table 3, which includes the conflict period, birth seasonality, and fixed locality effects. However, in this case, the conflict period is not divided by specific wars. Instead, we differentiate between localities that experienced the most severe battles and sieges (either at the time or in the immediate aftermath), the rest of the region during wartime without such intense conflicts, and peacetime. We use the overall peacetime period as the reference category. In total, we estimate five complete logistic regression models.

The results in Table 4 indicate that the reduction in sex ratios during wartime was not uniform across the region. The decline in sex ratios was concentrated in localities that experienced the most intense military actions. In these areas, the percentage of baptisms recorded as male dropped by over 1% during the year of the conflict and by nearly 2% in the following year, resulting in more female than male births overall during these periods.

Another key finding from Table 4 is the delay in the full manifestation of the battle’s impact. The most pronounced effect on sex ratios is observed in births occurring within the three months after the outbreak of the conflict (with a coefficient of -0.099). This means that the stress of war had its greatest effect on fetuses being in the first trimester of pregnancy. The effect of this stress was statistically significant in all trimesters of pregnancy but diminishes over the subsequent six months (-0.074) and is further reduced in the following nine months (-0.054). These results suggest that extreme stress caused by the proximity of conflict and the associated threats to life had a lingering effect, particularly in the immediate aftermath of major battles, likely to affect even mothers in advanced stages of pregnancy. Severe stress in pregnancy is most dangerous in the first trimester. But it is not just stress that affects the mother during pregnancy that has negative perinatal effects. Studies show that some stressors that mothers experience before pregnancy can affect the well-being of their offspring (Scorza et al., 2021). Pregnant women who experience post-traumatic stress in their childhood have a higher awakening cortisol response (Apanasewicz et al., 2022). Moreover, studies indicate intergenerational transmission of stress. The research demonstrates the potential impact of harsh conditions experienced by grandmothers on the perinatal outcomes of their grandchildren (Liczbińska & Králík, 2024). In the first three months after the outbreak of the conflict, the stress of the war itself may have played a role, as well as other stresses to which the mothers had been exposed prior to pregnancy.

**Table 4:** Probability of being registered at birth as a boy in Basque County (controlling for the localities more affected by the conflict), 19th century

Dependent variable: Sex (male = 1)		(1) Year	(2) Year + 1	(3) 3 months	(4) 6 months	(5) 9 months
Period	Peace	(ref.)				
	War	-0.002 (0.01)	-0.002 (0.01)	-0.001 (0.01)	-0.002 (0.01)	-0.002 (0.01)
	Localities directly impacted by the most intense war events	-0.042 (0.03)	-0.068** (0.03)	-0.098* (0.06)	-0.074* (0.04)	-0.054* (0.04)
Season of birth	Winter	(ref.)				
	Spring	-0.012** (0.01)	-0.012** (0.01)	-0.012** (0.01)	-0.012** (0.01)	-0.012** (0.01)
	Summer	0.007 (0.01)	0.007 (0.01)	0.007 (0.01)	0.007 (0.01)	0.007 (0.01)
	Fall	0.004 (0.01)	0.004 (0.01)	0.004 (0.01)	0.004 (0.01)	0.004 (0.01)
Control Year		Yes	Yes	Yes	Yes	Yes
Control Year squared		Yes	Yes	Yes	Yes	Yes
Locality Fixed Effects		Yes	Yes	Yes	Yes	Yes
	Sample size	1,204,860	1,204,860	1,204,860	1,204,860	1,204,860

Source: Complete parish registers of the Basque Country

Notes: *se* denotes robust standard error. * Statistical significance at 10% level, ** at 5% level. *** at 1% level. The same analyses, excluding the variables *year* and *year squared*, can be found in Table A.3 of the Appendix. For simplicity, the intercept is not reported

### By Duration of the Battle/Siege

Finally, since Table 4 demonstrated that the key factor driving changes in sex ratios at birth during premodern wartime was the proximity of battles, we now aim to determine whether the duration of those battles also had an effect. To address this, Table 5 builds on the regression models from Table 4, but categorizes all battles and sieges based on their duration: single-day events (including 11 battles and sieges), those lasting up to one week (2–7 days), including four of them, and those extending beyond one week (including 6 events).

**Table 5:** Probability of being registered at birth as a boy in Basque County (controlling for the localities more affected by the conflict and number of days of impact), 19th century

Dependent variable: Sex (male = 1)		(1) Year	(2) Year + 1	(3) 3 months	(4) 6 months	(5) 9 months
Period	Peace	(ref.)				
	War	-0.002 (0.01)	-0.002 (0.01)	-0.001 (0.01)	-0.002 (0.01)	-0.002 (0.01)
	Localities directly impacted by the most intense battles (1 day)	-0.045 (0.06)	-0.108 (0.09)	-0.053 (0.12)	-0.125 (0.08)	-0.113 (0.07)
	Localities (2–7 days)	-0.035 (0.29)	-0.204 (0.24)	0.566 (1.23)	0.460 (0.63)	0.532 (0.44)
	Localities (> 7 days)	-0.041 (0.04)	-0.058* (0.04)	-0.115* (0.07)	-0.059* (0.05)	-0.040 (0.04)
Season of birth	Winter	(ref.)				
	Spring	-0.012** (0.01)	-0.012** (0.01)	-0.012** (0.01)	-0.012** (0.01)	-0.012** (0.01)
	Summer	0.007 (0.01)	0.007 (0.01)	0.007 (0.01)	0.007 (0.01)	0.007 (0.01)
	Fall	0.004 (0.01)	0.004 (0.01)	0.004 (0.01)	0.004 (0.01)	0.004 (0.01)
Control Year		Yes	Yes	Yes	Yes	Yes
Control Year squared		Yes	Yes	Yes	Yes	Yes
Locality Fixed Effects		Yes	Yes	Yes	Yes	Yes
	Sample size	1,204,860	1,204,860	1,204,860	1,204,860	1,204,860

Source: Complete parish registers of the Basque Country

Notes: *se* denotes robust standard error. * Statistical significance at 10% level, ** at 5% level. *** at 1% level. The same analyses, excluding the variables *year* and *year squared*, can be found in Table A.4. of the Appendix. For simplicity, the intercept is not reported

The logistic regression results in Table 5 confirm, albeit with less robust findings compared to previous tables (due to the low number of events), that the impact of conflict on sex ratios was particularly pronounced when exposure to battle—and consequently to associated shortages and diseases—was prolonged. Notably, the only statistically significant negative coefficient exceeding − 0.100 across all models in this study is linked to births occurring during the conflict period or within three months afterward in localities that experienced military actions lasting more than one week (often extending to several months). Regardless of statistical significance, all conflicts—irrespective of their duration—are associated with a reduction in male births. The underlying causes of this decline are discussed in the following section.

## Discussion

Our analysis of Tables 2 and 3, and 4 demonstrates that premodern conflicts resulted in a significant reduction in sex ratios at birth, especially when battles were prolonged and perceived as nearby. All wars caused variations in demographic behavior, influencing deviations in sex ratios at birth by approximately 1%. However, the First Carlist War had the most profound impact. This effect was particularly pronounced in the main conflict zones, where sieges and major battles occurred, leading to a 3.2% deviation in sex ratios. The most substantial effects were observed during the three months following the events, with prolonged battles resulting in an average effect of nearly 7% on sex ratios at birth.

Orzack and colleagues (2015) examined the sex ratio at all stages of pregnancy, from conception to delivery. They found that the sex ratio at conception is equal, indicating no difference in the number of males and females conceived. Therefore, differences in the sex ratio at birth must be attributed to miscarriage during pregnancy. Their research indicates that the risk of miscarriage is generally higher for female foetuses compared to male ones. However, this risk varies depending on the stage of pregnancy: during the first week, male mortality is higher, whereas from the 10th to the 15th weeks, female mortality is higher. By around week 20, the mortality rate for male and female foetuses is approximately the same. Between the 28th and 35th weeks, the risk of death is higher for males. This suggests that male foetuses may have been more likely to be spontaneously aborted in the early stages of pregnancy.

Our findings align with the hypothesis of an excess miscarriage of male fetuses due to maternal stress (Byrne & Warburton, 1987; Hobel et al., 1999; James, 2015). Probably, between the 28th and 35th weeks (Orzack et al., 2015), as a consequence of higher rates of spontaneous male miscarriage under stressful circumstances (Barrett & Swan, 2015; Traylor et al., 2020). Male fetuses grow larger and require greater maternal resources, making them less adaptable to the stressful intrauterine environment, especially to food shortages. In contrast, female fetuses require fewer resources during development and may reduce their growth and maternal demands in response to stress (Sandman et al., 2006).

In both the Napoleonic War and the First Carlist War, we observe (as shown in Fig. 6) that the initial drop in sex ratios at the onset of the conflict is later offset by a recovery over time. This pattern is not observed during the Second Carlist War and is only barely present during the Third Carlist War. Moreover, in the regressions presented in Table 2, the impact of the Napoleonic War does not appear statistically significant, possibly due to this later compensatory effect. The reasons behind this evolution are not entirely clear. One possible explanation is that stress levels decrease over time as people adapt to living under conflict. Another plausible reason is that individuals with higher socioeconomic and biological status were in better conditions to have children—including a higher likelihood that the male head of household was not sent to the front—which could have made them less affected by the war’s impact due to their more favorable material conditions. In any case, further research is needed to fully understand the dynamics of sex ratio evolution during wartime—especially in longer and older conflicts—and to determine whether these patterns can be extrapolated to other historical or geographical contexts.

Our demographic analysis highlights that nearly all 19th-century wars, with the exception of the Second Carlist War, were associated with sharp declines in fertility in the Basque Country, a key factor in interpreting the evolution of sex ratios. A 10% reduction in mothers giving birth, particularly among specific socioeconomic or age groups, could partially explain these results. It is well established that maternal age, characteristics, and nutritional status affect the likelihood of male versus female births as well as the probability of twin births (Rosenfeld & Roberts, 2004; Rueness et al., 2012; Marco-Gracia & Beltrán Tapia, 2022; Marco-Gracia, 2024). Male fetuses are more sensitive to famine or food shortages than female ones, which could explain the decline in SRB (MacIntyre, 2002).

As supported by existing literature, the First Carlist War (1833–1840) had the most significant demographic impact on the Basque region (Ilacqua, 2024). This war was marked by intense military activity, resulting in widespread social and economic upheaval. Our findings show that the First Carlist War had the highest mortality increase (over 10%), the largest decline in fertility (nearly 10%), and a sharp drop in marriage rates (approximately 50%) compared to the pre-war period. These observations confirm that any study of premodern wars in the Basque Country must take the First Carlist War as a reference point.

Additionally, the seasonality effects observed in all regression models for the Basque Country highlight the significance of springtime reductions in sex ratios during the 19th century, reflecting a higher proportion of female births relative to male births. This seasonal fluctuation may be attributed to environmental factors such as temperature and photoperiod, which are known to influence reproductive patterns (Kumari & Rao, 1982). Research on populations from the past shows that ambient temperature, similar to psychological stress, hunger, malnutrition, and social and economic factors, influenced the viability of a fetus, and suggests a positive impact of warm months on the conception and fetal development and the increase in the ratio of live-born boys to girls (Liczbińska et al., 2024). Longer daylight hours and higher temperatures may impact hormonal balances, leading to a higher conception rate of female offspring. Sociocultural factors, including seasonal labor patterns and nutritional status, likely also played a role in shaping these seasonal variations (Dahlberg & Andersson, 2018).

This study has several limitations, such as relying on baptismal records instead of birth records, which might exclude live births not baptized, even though the time between birth and baptism was short due to Catholic Church directives. Furthermore, the database’s structure prevented the use of family reconstitution methods, which could have provided additional insights. Nevertheless, this research, based on over one million individual microdata entries, offers significant contributions to understanding preindustrial wars and their impact on sex ratios at birth.

## Conclusions

This study emphasizes the profound impact of 19th-century wars in the Basque Country on sex ratios at birth. Through the analysis of an extensive dataset comprising nearly 1.2 million baptismal records, we present compelling evidence that premodern wars, even those involving relatively limited destruction compared to modern conflicts, had discernible effects on biological and demographic outcomes.

Among the conflicts that affected the Basque Country during the 19th century, the First Carlist War (1833–1840) emerges as the most significant, producing notable deviations in sex ratios at birth. War also left a demographic imprint, with variations in sex ratios being particularly pronounced in areas that experienced sieges and major battles. These effects, attributed to maternal stress and hormonal changes during pregnancy, were temporally distributed and became most evident several months after the conflict events.

Our findings highlight the exceptional influence of prolonged battles, which exerted the greatest impact on sex ratios at birth. This led to an average decline in sex ratios during the period of the battle and the three months following it by 14 boys, dropping from an average of 106 boys born per 100 girls during peacetime to just 92.2 boys per 100 girls during those months.

Overall, this research demonstrates that the demographic consequences of premodern warfare extend beyond immediate mortality and population displacement, encompassing more nuanced biological responses. These findings contribute to a deeper understanding of the complex interplay between conflict and human biology, especially human reproduction in past societies.

## Electronic Supplementary Material

Below is the link to the electronic supplementary material.

Supplementary Material 1

## Data Availability

This research is based on a dataset compiled from all existing parish archives in the Basque Country for the 19th century. These records are publicly available, having been digitized and transcribed by the regional government of the Basque Country. They can be accessed individually via their website or upon justified request to the institution (director of the archive: Josebe-alonso@euskadi.eus). The data are not subject to data protection laws, as more than 100 years have elapsed since the last recorded entry. https://www.artxibo.euskadi.eus/webartxi00-container/es/ad53aArchivoHistoricoWar/sacramentales/maintSimple.

## Footnotes

1 Bordered by France to the north and integrated into Spain to the south, the region is distinguished by its unique language, Euskara, which stands as a linguistic isolate, and its deeply rooted cultural identity developed over centuries (Woodworth, 2007).

2 https://www.artxibo.euskadi.eus/webartxi00-container/es/ad53aArchivoHistoricoWar/sacramentales/maintSimple [Last visit: November 19 2024].

3 Unfortunately, a more in-depth analysis is not currently feasible because the individuals are not linked to the various events in their lives.

4 Names that could not be reliably assigned to a specific sex were excluded from the analysis, but they account for less than 1% of the total. It is true that transcription errors may have occurred, especially since in many cases the only difference between male and female names is the final letter. However, we have no reason to believe that one gender was systematically overrepresented compared to the other, particularly not during specific periods of conflict when compared to the rest of the century.

5 In the Catholic world—particularly in a deeply conservative region like the Basque Country—it was considered essential for newborns to receive baptism in order to access the Catholic heaven. For this reason, we consider it acceptable to use the number of baptisms as a proxy for the number of births, as baptisms were typically performed shortly after birth, and urgently when necessary. Nevertheless, some births may be missing from the records, mainly due to unexpected deaths occurring within the first few hours of life. However, we do not believe that such early mortality could account for the patterns observed in this study, even if it might have had a minor impact on the results. Sudden shifts in sex ratios are unlikely to be explained by excess mortality in the brief interval between birth and baptism (many times the birth-baptism gap was under 24-hours).

6 Year and year squared have been included to account for changes in sex ratios at birth over the century, driven by improvements in living standards and other individual and environmental factors. Additionally, all models have been replicated in the appendix without incorporating these variables.

7 In Table 2, we have included only those war years in which the conflict spanned the entire calendar year, from January to December, excluding the first and last years of each war as they were incomplete. In Table A.2 of the Appendix, the same analysis is replicated but covering the full period from the first to the last day of the conflict, confirming the results of Table 2.

8 In Table 3, we use the period before the Napoleonic War as the reference point, while in Table 4, we take all peacetime periods as a whole. Any chosen reference period can be debated, as it may or may not fully reflect the desirable parameters given the social, cultural, or biological changes that can occur over the course of a century. However, to ensure the robustness of our results, we replicated the models by separating each conflict and using, for each war, the six (and twelve) months preceding it as the reference period. The results remained similarly significant and pointed in the same direction, thus confirming the robustness of the analysis. Note also that all specifications control for time trends (year and year squared).

9 This approach allows us to effectively manage the birth/baptismal database in its current form.

10 The selection of the most significant battles was based on the following key works on Carlism and the history of Spain: Oyarzun, 1944; Ferrer, 1958; Bullón de Mendoza, 1991; Cobo & Pros Mani, 2009; Barraycoa, 2019.
