# Supplementary material for: Did Premodern Wars Impact Sex Ratios at Birth? The Case of 19th-Century Basque Country
Source: Hum Nat. 2025 Jul 9;36(2):281–306. doi: 10.1007/s12110-025-09496-8 (PMC12417252; doi:10.1007/s12110-025-09496-8)
Supplement: Supplementary file 1 — Supplementary Material 1 [file 12110_2025_9496_MOESM1_ESM.docx]

**Appendix**

Table A.1. Probability of being registered at birth as a boy in Basque County, 19^th^ century

| Dependent variable: Sex (male=1) | | (1) | (2) | (3) |
| --- | --- | --- | --- | --- |
| Historical event | Before Napoleonic War | (ref.) |  |  |
|  | Napoleonic War | -0.003  (0.01) | -0.003  (0.01) | -0.010  (0.01) |
|  | First Carlist War | -0.021**  (0.01) | -0.020**  (0.01) | -0.034**  (0.01) |
|  | Second Carlist War | -0.009  (0.01) | -0.010  (0.01) | -0.016  (0.02) |
|  | Third Carlist War | -0.024**  (0.01) | -0.024**  (0.01) | -0.018  (0.02) |
|  | Interwar periods | -0.009  (0.01) | -0.009  (0.01) | -0.012  (0.01) |
|  | After Third Carlist War | -0.010  (0.01) | -0.010  (0.01) | -0.012  (0.01) |
| Season of birth | Winter | (ref.) |  |  |
|  | Spring |  | -0.021***  (0.01) | -0.016**  (0.01) |
|  | Summer |  | 0.005  (0.01) | 0.007  (0.01) |
|  | Fall |  | 0.002  (0.01) | 0.003  (0.01) |
| Locality Fixed Effects | | No | No | Yes |
|  | Intercept | 0.072***  (0.01) | 0.076***  (0.01) | 0.063**  (0.03) |
|  | Sample size | 1,209,058 | 1,204,861 | 1,204,860 |

Source: Complete parish registers of the Basque Country.

Notes: *se* denotes robust standard error. * Statistical significance at 10% level, ** at 5% level. *** at 1% level.

Table A.2. Probability of being registered at birth as a boy in Basque County, 19^th^ century.

| Dependent variable: Sex (male=1) | | (1) | (2) | (3) |
| --- | --- | --- | --- | --- |
| Historical event | Before Napoleonic War | (ref.) |  |  |
|  | Napoleonic War | 0.001  (0.01) | 0.001  (0.01) | 0.001  (0.01) |
|  | First Carlist War | -0.017*  (0.01) | -0.017*  (0.01) | -0.033**  (0.01) |
|  | Second Carlist War | -0.013  (0.01) | -0.014  (0.01) | -0.019  (0.02) |
|  | Third Carlist War | -0.007**  (0.01) | -0.008**  (0.01) | -0.010  (0.01) |
|  | Interwar periods | -0.004  (0.01) | -0.005  (0.01) | -0.005  (0.01) |
|  | After Third Carlist War | -0.009  (0.01) | -0.009  (0.01) | -0.012  (0.01) |
| Season of birth | Winter | (ref.) |  |  |
|  | Spring |  | -0.020***  (0.01) | -0.016**  (0.01) |
|  | Summer |  | 0.004  (0.01) | 0.006  (0.01) |
|  | Fall |  | 0.001  (0.01) | 0.003  (0.01) |
| Locality Fixed Effects | | No | No | Yes |
|  | Intercept | 0.070***  (0.01) | 0.074***  (0.01) | 0.061**  (0.03) |
|  | Sample size | 1,209,058 | 1,204,861 | 1,204,860 |

Source: Complete parish registers of the Basque Country.

Notes: *se* denotes robust standard error. * Statistical significance at 10% level, ** at 5% level. *** at 1% level.

Table A.3. Probability of being registered at birth as a boy in Basque County (controlling for the localities more affected by the conflict), 19^th^ century.

| Dependent variable: Sex (male=1) | | (1)  Year | (2)  Year+1 | (3)  3 months | (4)  6 months | (5)  9 months |
| --- | --- | --- | --- | --- | --- | --- |
| Period | Peace | (ref.) |  |  |  |  |
|  | War | 0.002  (0.01) | 0.002  (0.01) | 0.003  (0.01) | 0.002  (0.01) | 0.002  (0.01) |
|  | Localities directly impacted by the most intense war events | -0.043  (0.03) | -0.068**  (0.03) | -0.099*  (0.06) | -0.074*  (0.04) | -0.054*  (0.04) |
| Season of birth | Winter | (ref.) |  |  |  |  |
|  | Spring | -0.012**  (0.01) | -0.012**  (0.01) | -0.012**  (0.01) | -0.012**  (0.01) | -0.012**  (0.01) |
|  | Summer | 0.007  (0.01) | 0.007  (0.01) | 0.007  (0.01) | 0.007  (0.01) | 0.007  (0.01) |
|  | Fall | 0.004  (0.01) | 0.004  (0.01) | 0.004  (0.01) | 0.004  (0.01) | 0.004  (0.01) |
| Locality Fixed Effects | | Yes | Yes | Yes | Yes | Yes |
|  | Intercept | 0.050***  (0.03) | 0.051***  (0.03) | 0.050***  (0.03) | 0.050***  (0.03) | 0.050***  (0.03) |
|  | Sample size | 1,204,860 | 1,204,860 | 1,204,860 | 1,204,860 | 1,204,860 |

Source: Complete parish registers of the Basque Country.

Notes: *se* denotes robust standard error. * Statistical significance at 10% level, ** at 5% level. *** at 1% level.

Table A.4. Probability of being registered at birth as a boy in Basque County (controlling for the localities more affected by the conflict and number of days of impact), 19^th^ century.

| Dependent variable: Sex (male=1) | | (1)  Year | (2)  Year+1 | (3)  3 months | (4)  6 months | (5)  9 months |
| --- | --- | --- | --- | --- | --- | --- |
| Period | Peace | (ref.) |  |  |  |  |
|  | War | 0.002  (0.01) | 0.002  (0.01) | 0.003  (0.01) | 0.002  (0.01) | 0.002  (0.01) |
|  | Localities directly impacted by the most intense battles (1 day) | -0.045  (0.06) | -0.109  (0.09) | -0.053  (0.12) | -0.125  (0.08) | -0.113  (0.07) |
|  | Localities (2-7 days) | -0.037  (0.29) | -0.206  (0.24) | 0.564  (1.23) | 0.458  (0.63) | 0.530  (0.44) |
|  | Localities (>7 days) | -0.042  (0.04) | -0.059*  (0.04) | -0.116*  (0.07) | -0.060*  (0.05) | -0.041  (0.04) |
| Season of birth | Winter | (ref.) |  |  |  |  |
|  | Spring | -0.012**  (0.01) | -0.012**  (0.01) | -0.012**  (0.01) | -0.012**  (0.01) | -0.012**  (0.01) |
|  | Summer | 0.007  (0.01) | 0.007  (0.01) | 0.007  (0.01) | 0.007  (0.01) | 0.007  (0.01) |
|  | Fall | 0.004  (0.01) | 0.004  (0.01) | 0.004  (0.01) | 0.004  (0.01) | 0.004  (0.01) |
| Locality Fixed Effects | | Yes | Yes | Yes | Yes | Yes |
|  | Intercept | 0.050***  (0.03) | 0.051***  (0.03) | 0.050***  (0.03) | 0.051***  (0.03) | 0.051***  (0.03) |
|  | Sample size | 1,204,860 | 1,204,860 | 1,204,860 | 1,204,860 | 1,204,860 |

Source: Complete parish registers of the Basque Country.

Notes: *se* denotes robust standard error. * Statistical significance at 10% level, ** at 5% level. *** at 1% level.
